# Dark Joints in Vulcanite Plates—How to Prevent Same

**Published:** 1896-09

**Authors:** 


ARTICLE IX.

### DARK JOINTS IN VULCANITE PLATES—HOW TO PREVENT SAME.

There is a great deal of trouble among those who do vulcanite plate work, to avoid dark joints. I submit, herewith, my method of making vulcanite plates, which, if followed out, I think will prevent dark joints to a considerable degree.

Everything should be scrupulously clean throughout the entire process. To commence with, the grinding of the teeth should be done under a magnifying glass, in order to have a perfect fitting joint. I use two corundum wheels. The first, a coarse wheel, to knock off the extra gum; the second, a finer wheel, which enables me to make a nice, smooth joint. For waxing up the plates, I use paraffine and wax, which by experience I found superior to straight bee's wax, as the work can be kept much cleaner. Now, I have my teeth ground up and waxed. The next process is to chill the wax, removing each block, commencing at the molar block. Be careful not to damage the wax. After all of the blocks have been removed, then with a sharp knife shave off the feather edge of the wax, above the place where the pins come, as high as you want the rubber to come after completion of plate. Before replacing the teeth in the wax, put them in boiling water, in order to remove surplus wax; wash with soap and water so as to get them perfectly clean and trim the plate. Then, mix a little of any of the cements to a thin mixture, and place a small quantity on the joint surface, which will thoroughly seal the joints. Next, finish up the wax as you want the rubber when done. The more pains taken in waxing, the less work in removing plate from vulcanizer. If everything is kept clean up to this point, you will next be ready to flask. You will flask as usual, except after varnishing, instead of using oil, you will find soap-water much better. Use as little oil as possible. The oil, in my opinion, has a great deal to do with dark joints. Now, you have the plate flasked. Lay it aside for four or five hours in order to give the cement time to harden. Separate flask and flood with hot water, so as to remove all particles of wax that may have been left by heating flask for separation. After this is accomplished, pack your rubber. Use a little more rubber than wax. Place over the rubber a piece of thin, wet cloth. Then put your flask together and place it in boiling water. After remaining ten minutes, put under screw press, with tight pressure Separate and remove the cloth, trim off the excess of rubber, close and vulcanize.

If the above instructions are followed, I think dark joints will be avoided.—*Items of Interest.*

